# Supplementary material for: Assessment of Untargeted Metabolomics by Hydrophilic Interaction Liquid Chromatography−Mass Spectrometry to Define Breast Cancer Liquid Biopsy-Based Biomarkers in Plasma Samples
Source: Int J Mol Sci. 2024 May 7;25(10):5098. doi: 10.3390/ijms25105098 (PMC11120904; doi:10.3390/ijms25105098)
Supplement: Supplementary file 1 [file ijms-25-05098-s001.zip › ijms-2963287-supplementary.pdf]

Research Article

Supplementary Material

Title: Assessment of Untargeted Metabolomics by Hydrophilic Interaction Liquid Chromatography – Mass Spectrometry to Define Breast Cancer Liquid Biopsy-Based Biomarkers in Plasma Samples

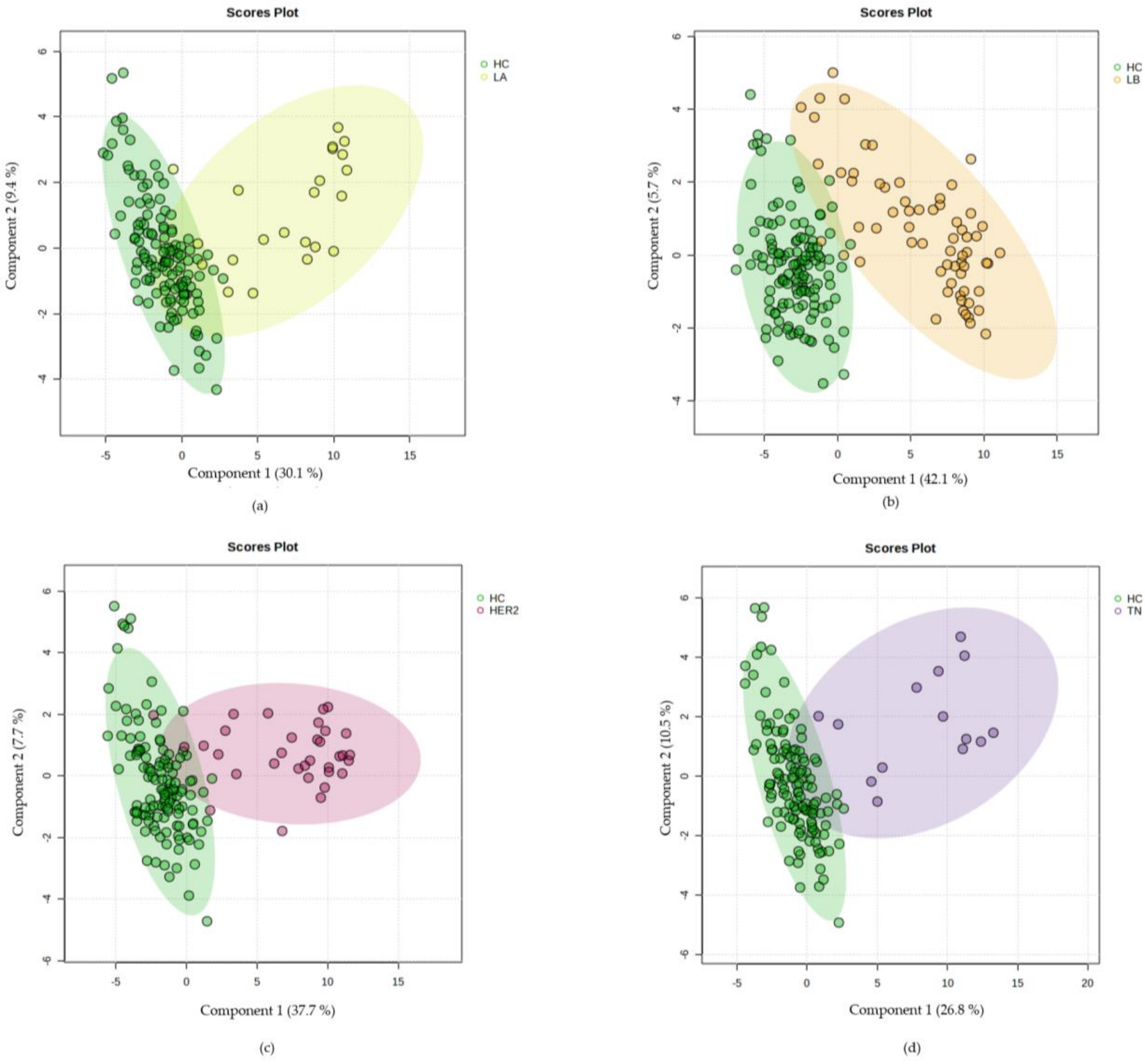

Figure S1. The PLS-DA score plots based on the LC-HRMS of plasma samples from BC suggests metabolome differences according to the different molecular subtypes in comparison with the HC group: (a) luminal A (LA), (b) luminal B (LB), (c) human epidermal growth factor receptor 2 positive (HER2), (d) triple negative (TN).

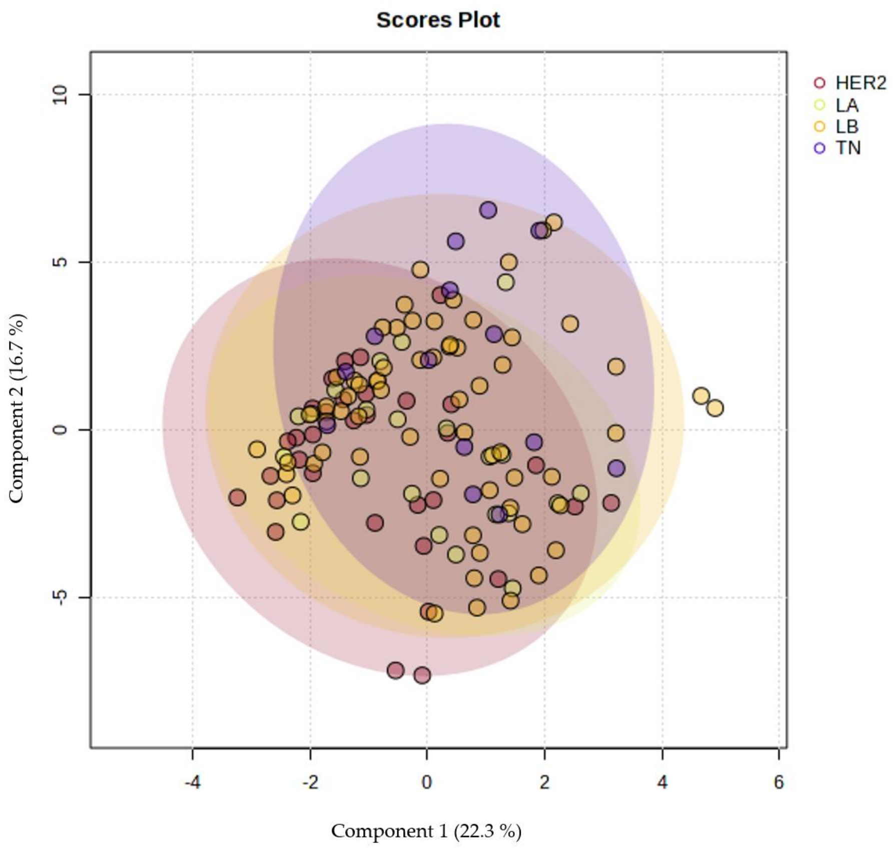

Figure S2. The PLS-DA score plots from the four molecular subtypes shows a clustering of the luminal samples in the middle while the human epidermal growth factor receptor 2 positive (HER2) and triple negative (TN).

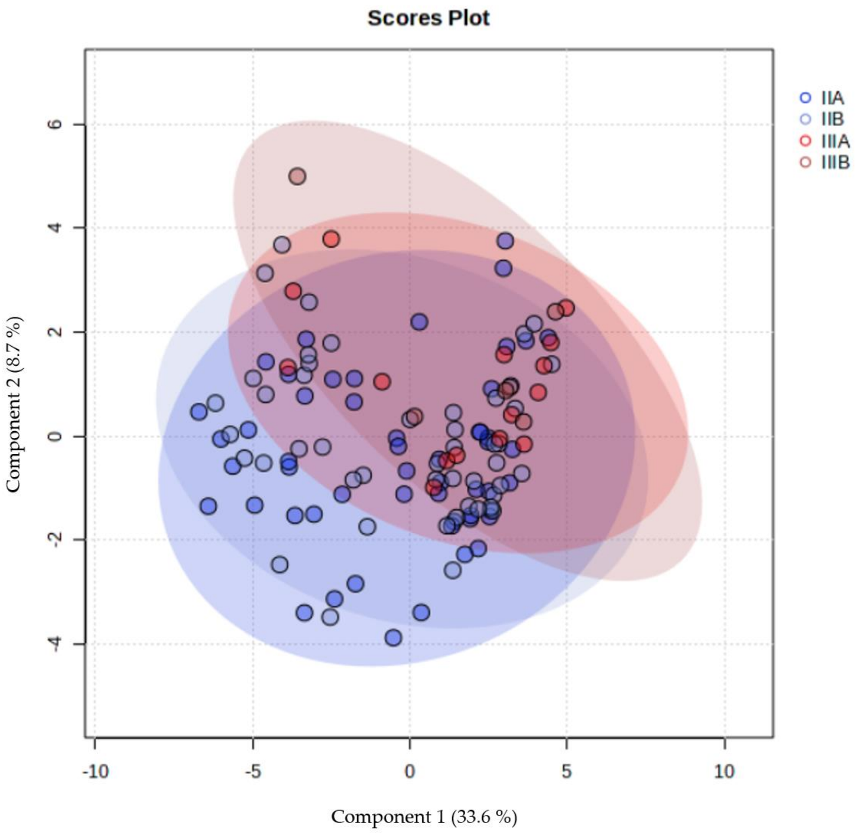

Figure S3. The PLS-DA score plots from BC samples stratified by stages shows a clustering according to the stages II (A- dark blue, B- light blue circles) and III (A – dark red, B – light red circles).

Table S1. Statistical validation of the PLS-DA models based on the comparison of breast cancer (BC) molecular subtypes to healthy controls (HC).

PLS-DA comparison	Explained Variation (%)	# Components	Accuracy	R^2	Q^2
**LA-HC**	56.4	5	0.974	0.850	0.688
**LB-HC**	61.1	5	0.968	0.901	0.833
**HER2-HC**	60.7	5	0.969	0.886	0.767
**TN-HC**	53.1	5	0.979	0.883	0.677

LA: luminal A; LB: luminal B; HER2: human epidermal growth factor receptor 2; TN: triple negative; HC: healthy controls; #: number; R^2 and Q^2 parameters showed that no over-fittingwas observed, and these models are acknowledged for successful discernment between HC and BC patients.

Table S2. List of metabolites that significantly differed between breast cancer (BC) and healthy controls (HC) samples and their respective p--value, fold change, VIP score and univariate values of AUC.

m/z	R.T (min)	p-value (FDR)	Fold Change (BC/HC)	VIP	AUC
1045.0609	3.16	4.13×10^{-29}	0.12	1.11	0.88

988.1027	3.19	2.01×10^{-55}	0.14	2.36	0.93
746.3442	3.19	1.13×10^{-48}	0.14	2.33	0.93
992.1087	3.19	4.20×10^{-54}	0.15	2.35	0.93
956.1536	3.2	1.74×10^{-53}	0.15	2.17	0.93
1020.1459	3.16	1.76×10^{-54}	0.15	2.22	0.93
992.1087	3.19	1.37×10^{-54}	0.16	2.40	0.93
1022.1394	3.17	1.74×10^{-53}	0.16	2.38	0.93
974.1329	3.17	2.34×10^{-51}	0.17	2.05	0.92
1026.1296	3.16	1.19×10^{-52}	0.17	2.31	0.93
1024.1411	3.16	9.33×10^{-53}	0.17	2.45	0.93
1008.1211	3.16	9.33×10^{-53}	0.17	2.37	0.94
1030.1737	3.14	2.09×10^{-51}	0.18	2.31	0.93
89.0273	1.37	2.25×10^{-28}	0.18	1.06	0.88
886.2949	3.15	7.66×10^{-44}	0.18	2.21	0.93
1032.1746	3.15	1.22×10^{-49}	0.19	2.21	0.92
1040.1896	3.15	1.98×10^{-45}	0.22	2.10	0.91
1038.1741	3.15	8.66×10^{-46}	0.22	2.17	0.91
1044.1953	3.16	5.28×10^{-44}	0.22	2.07	0.91
892.3343	3.15	3.56×10^{-40}	0.23	2.25	0.91
635.4445	1.03	4.63×10^{-15}	0.23	1.03	0.78
808.3709	1.45	4.12×10^{-14}	0.23	1.02	0.80
816.4075	3.13	4.90×10^{-38}	0.24	2.15	0.91
1046.1901	3.15	7.32×10^{-40}	0.24	1.97	0.90
976.2954	3.14	4.67×10^{-35}	0.28	1.90	0.88
1054.2285	3.14	3.55×10^{-35}	0.28	1.92	0.87
696.7823	3.34	6.02×10^{-30}	0.29	1.87	0.94
902.3649	3.14	1.40×10^{-31}	0.30	2.01	0.86
742.4737	3.15	2.49×10^{-26}	0.31	2.35	0.89
828.4322	3.15	6.87×10^{-26}	0.33	1.94	0.86
1060.2524	3.13	3.77×10^{-28}	0.36	1.68	0.82
910.3963	3.12	3.77×10^{-26}	0.37	1.73	0.82
834.5387	3.36	9.43×10^{-20}	0.45	1.00	0.78
1068.2847	3.13	7.28×10^{-21}	0.47	1.51	0.76
870.6637	3.48	1.75×10^{-44}	20.97	1.30	0.93
870.6576	3.27	7.66×10^{-43}	22.85	1.28	0.93
810.7045	3.34	1.32×10^{-45}	39.07	1.43	0.93
812.6938	3.4	5.76×10^{-49}	50.31	1.50	0.94
642.7599	3.15	1.18×10^{-44}	55.71	1.43	0.93
712.7536	3.18	2.93×10^{-42}	61.80	1.41	0.91
880.6902	3.22	1.03×10^{-48}	71.83	1.68	0.93
824.8018	3.15	9.98×10^{-47}	88.06	1.84	0.93
630.7895	3.34	8.00×10^{-48}	93.27	1.72	0.93
710.7503	3.16	4.48×10^{-50}	94.34	1.63	0.94
836.6892	3.14	8.29×10^{-48}	112.18	1.74	0.94
698.7835	3.34	3.82×10^{-49}	141.57	1.91	0.94
822.7321	3.34	9.43×10^{-49}	143.02	1.82	0.94
888.7159	3.34	4.82×10^{-47}	169.94	1.97	0.93
764.7638	3.22	2.62×10^{-46}	262.58	1.99	0.93
762.7784	3.34	9.30×10^{-49}	348.90	1.99	0.95

m/*z*: mass/charge ratio; R.T: retention time; FC: fold change > 2 indicates that the average normalized peak area ratio in breast cancer (BC) samples is larger than that in healthy controls (HC); FC < 0.5 indicates that the average normalized peak area ratio in HC is larger than that in BC samples; VIP: variable of importance in projection; AUC: area under the receiver-operating characteristic curve.
